# Lipschütz Genital Ulceration as Initial Manifestation of Primary Sjögren's Syndrome

**DOI:** 10.1155/2018/3507484

**Published:** 2018-06-03

**Authors:** Filipa de Castro Coelho, Maria Amaral, Lúcia Correia, Maria João Nunes Campos, Tereza Paula, Augusta Borges, Jorge Borrego

**Affiliations:** ^1^Gynecology Department, Hospital Dr. Nélio Mendonça, Serviço de Saúde da Região Autónoma da Madeira, EPE, Funchal, Portugal; ^2^Internal Medicine Department, Maternidade Dr. Alfredo da Costa, Centro Hospitalar Lisboa Central, Lisboa, Portugal; ^3^Gynecology Department, Instituto Português de Oncologia de Lisboa Francisco Gentil, EPE, Lisboa, Portugal; ^4^Gynecology Department, Maternidade Dr. Alfredo da Costa, Centro Hospitalar Lisboa Central, Lisboa, Portugal

## Abstract

Genital ulcers are challenging to any clinician and causes transcend many specialties. Skin ulceration in patients with primary Sjögren's syndrome is infrequent but an established feature of cutaneous involvement. Although gynecological symptoms, such as vulvovaginal dryness, dyspareunia, and pruritus, are common in women with primary Sjögren's syndrome, patients affected by vulvar ulcers are unknown. We describe an exceptional case of necrotic aphthous-type vulvar ulceration as initial presentation of primary Sjögren's syndrome that was possibly triggered by an infectious agent. Successful healing was achieved with oral corticosteroids, despite some loss of labia* minora* and labia* majora* as sequelae of the necrotizing process. Reactive acute genital ulcers (Lipschütz ulcers) should be considered as a possible manifestation of many autoimmune/inflammatory disorders, beyond the classic associations such as Behçet's syndrome or Crohn's disease.

## 1. Introduction

Primary Sjögren's syndrome (pSS) is an autoimmune inflammatory disorder, with a higher incidence in female patients. Extraglandular manifestations may result from lymphocytic infiltration, generation of pathogenic autoantibodies-mediated mechanisms, or vasculitis [1]. Cutaneous vasculitis is generally thought of as being palpable purpura, but other infrequent types of lesions can occur, such as ulcers or ischemic lesions [1, 2]. Vascular findings of pSS often occur on the legs, although a perivascular lymphocytic infiltrate has been observed in the underlying vaginal stroma of women with Sjögren's syndrome (SS) [3].

Cutaneous disorders serve as a reflex of systemic involvement. Autoimmune conditions can be triggered by infectious agents, which can also determine its clinical manifestations [4]. Lipschütz ulcers (LUs) or reactive nonsexually related acute genital ulcers (RNSAGU) are often described as a clinical sign of an immune response to an underlying infection. LU's and pSS's pathogenesis share common infectious triggers across literature [4, 5]. Thus, a role for infection in both conditions is suggested. We present a case of a woman with Lipschütz genital ulceration as initial manifestation of pSS in the context of an infectious disease.

## 2. Case

**Figure 1:**
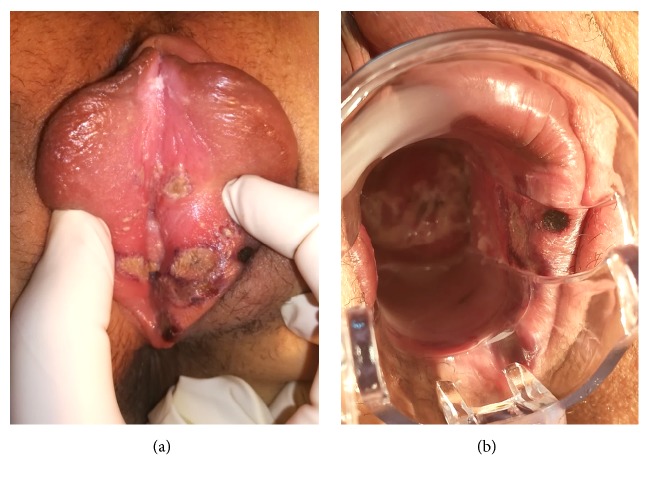
(a) Vulvar swelling and shallow “kissing” ulcers with necrotic debris and sharply demarcated borders, 2 mm-20 mm diameter, on labia minora and majora. (b) Ulcers on the bilateral labia, lower vagina, and cervix.

**Figure 2:**
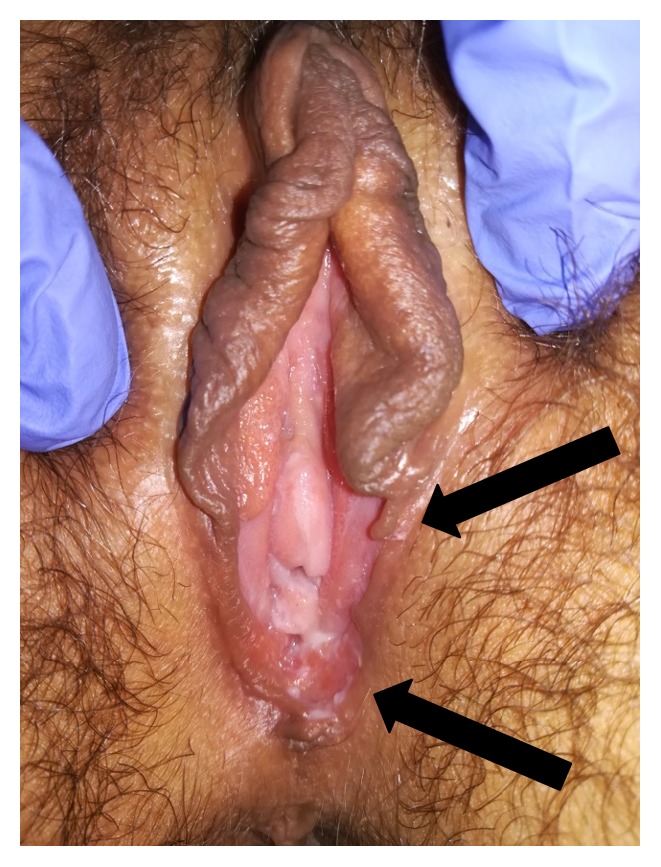
Complete resolution of the vulvar lesions with partial loss of left labia* minora* and* majora* (black arrows).

A 26-year-old-woman, with history of labial herpes and asthmatic bronchitis, presented with bilateral retroocular pain, odynophagia, fever, vaginal discomfort and vulvar ulcers. The ulcers continued to progress despite treatment with nonsteroidal anti-inflammatory drugs and valacyclovir, prescribed at the primary healthcare site. Two days later, after initial consultation at our emergency room, she was admitted immediately at the Vulvar Clinic of our institution, with increasing vulvar pain, without other symptoms. Physical examination of the vulva showed extended vulvar oedema and* kissing pattern* ulcers on labia* minora* and majora, vagina and cervix (Figure 1). Inguinal lymph nodes were also bilaterally swollen. The patient denied the use of other medications and sexual activity in more than 6 months. First blood tests only showed C-reactive protein 12.35 mg/dL (normal: <0.5 mg/dL). Serologies for herpes virus 1 and 2, Ebstein-Barr virus (EBV; IgG+), cytomegalovirus,* mycoplasma pneumoniae*, parvovirus B19, toxoplasmosis, rubella, hepatitis, human immunodeficiency virus, and syphilis (using the Venereal Disease Research Laboratory test) were negative. After this, a multidisciplinary approach was performed. When directly asked, she complained about mild eye dryness and she often felt a discomfort of dry mouth. There was no familial history of autoimmune diseases, but her father had some episodes of oral aphthosis. Immunological examination was positive for rheumatoid factor (RF – 22.3 UI; normal: <15 UI), anti-nuclear antibodies [ANA (speckled, titer 1:320)] and antibodies to SSA/Ro (SSA 3+/ Ro52KD 3+)—initial screening step of ANA by indirect immunofluorescence on HEp-2 cells (*Euroimmun ***®***, Germany)*; autoantibodies confirmation assay by line immunoblot (*ANA profile 3 - Euroimmun ***®***, Germany)*—antibodies detected on strips were evaluated semiquantitatively (negative, 1+, 2++, and 3+++). Anti-SSB/La, anti-RNP, anti-Sm, anti-dsDNA, antineutrophil cytoplasmic, anticardiolipin, and anti-beta(2)-glycoprotein1 antibodies were negative. Serum C3 level was 1.79 g/L (normal: 0.9-1.8 g/L) and C4 level was 0.31 g/L (0.1-0.4 g/L). Immunoglobulins (IgG, IgM, IgA) were measured and a high IgG level was found (20.50 g /L; normal: 7-16 g/L). Erythrocyte sedimentation rate was also high (45 mm/h; normal: <16 mm/h). Lupus anticoagulant and HLA-B27 were both negative. Ophthalmological evaluation was refused by the patient. Labial salivary gland (LSG) biopsy revealed focal lymphocytic sialadenitis (FLS), with a focus score (FS) =1 (per 4 mm2) obtained by four LSGs (3-5 mm). No other histopathological features were reported. The patient was diagnosed as having pSS on the basis of dry eyes and dry mouth, positive anti-SSA/Ro antibody, and typical histopathologic abnormalities on LSG biopsy. Prednisolone 20 mg/day was prescribed and vulvar healing appeared within 2 weeks with partial loss of left labia (Figure 2).

## 3. Discussion

pSS occurs in a primary form not associated with other well-defined autoimmune disease. Current consensus classification criteria for pSS by American College of Rheumatology and the European League Against Rheumatism (EULAR) set this diagnosis in our patient who meets the inclusion criteria (sicca symptoms) and has a score ≥4 based on anti-SSA/Ro antibody positivity and FLS (each scoring 3) [2].

A LSG biopsy is considered positive if minor salivary glands demonstrate FLS, with a FS of 1 or more (defined as several lymphocytic foci, containing more than 50 lymphocytes per 4 mm2 of glandular tissue) [6, 7]. Experts recommendations also propose to obtain a minimum of four LSGs, unless these are small (2 mm). [7] We have adopted this standardised consensus guidance for the use of LSG histopathology in the classification of pSS. FLS cannot be attributed when morphologic patterns of chronic inflammation such as nonspecific chronic sialadenitis and sclerosing chronic sialadenitis occur in LSG biopsy specimens [6–8]. Our histopathological assessment did not find these patterns. A FS count of 1 was present and this reflects SS autoimmunity [6]. We opted not to perform imaging tests. Future modifications of the present-day criteria may arise with the adoption of new diagnostic tests, such as salivary gland ultrasonography or the use of magnetic resonance imaging for a more precise, noninvasive diagnostic approach to SS. Yet, EULAR consensus guidance reinforces LSG as a key marker of pSS [2, 7, 8]. And further validation studies are required before imaging modalities are included in a new set of classification criteria for pSS [7, 8].

This chronic autoimmune inflammatory disorder yields multiple clinical expressions, including exocrine gland involvement and extraglandular disease features. Among extraglandular manifestations, cutaneous vasculitis may manifest as ulcers [1, 2]. Compared to the patients without vasculitis, affected patients had a higher prevalence of positive ANA, anti-Ro/SS-A antibodies, and RF [1, 2]. These were findings in our patient.

LUs,* ulcus vulvae acutum* and RNSAGU, are synonymous of a distinct aphthous-type ulcers classified either as an idiopathic process or as secondary to other diseases such as Crohn's, Behçet's, and various infectious conditions [9, 10]. The precise aetiology and pathogenesis remain unclear; however, LUs are described as a result from an exuberant immune response to an infection [5, 10]. Most cases do not have an identifiable pathogen [5, 10]. Reactive acute genital ulcers present with fever, malaise and inguinal lymphadenopathy, resembling our patient. The main vulvar symptom is pain, sometimes with characteristic “kissing pattern” and notable oedema of labia. Necrosis is a complication previously described [10]. LUs are typically located on labia* minora*, but our patient also had lesions on the labia* majora*, lower vagina and cervix. Time to full healing was coincident with previous reports [5]. Retrospective studies emphasized that LUs should not be seen as a young virginal women exclusive vulvar dermatoses [5, 10]. It is mandatory the exclusion of sexually and nonsexually infections, drug reactions and autoimmune/inflammatory conditions, including Crohn's and Behçet's disease [5, 9, 10]. The authors could not find any report regarding pSS as a systemic illness to exclude. Treatment is supportive, but if a systemic disease is suspected, the multidisciplinary approach is appropriate, and treatment should be targeted to the specific etiology.

Similar to LUs, the underlying cause of pSS remains obscure. Infectious agents (viruses, bacteria, parasites, and fungi) are thought to trigger inflammation, autoantibody production and immune-mediated tissue injury [1]. LU's and pSS's pathogenesis has its focus in the genetics–autoimmunity–infection triad across scientific literature. Recent studies have reemphasized the role of molecular mimicry in which genetically predisposed individuals may make immune responses to self-antigens by immune mistake. This model has been applied to several pathogens, with the EBV being the paradigmatic example, but the spectrum of possible microorganisms is getting wider [1, 4, 5]. In this report, an infectious cause well-grounded on clinical and laboratory findings (without an aetiological diagnosis) was identified as the probable trigger for the final diagnosis of Sjögren's syndrome-associated Lipschütz genital ulceration.

Dyspareunia, vulvovaginal dryness and pruritus are common symptoms in pSS women [3]. To our knowledge, there are no previous reports describing acute vulvar ulcers as a cutaneous manifestation of pSS. The wide diversity of cutaneous processes, both vasculitic and nonvasculitic, observed in pSS patients, suggests that skin involvement in this condition reflects systemic involvement and manifests itself in different clinical forms [1, 2].

Vaginal ulcers completely resolved with oral prednisolone, but partial loss of left labia was observed. SS may be a predisposing factor for the impaired healing process [11].

No genital biopsies were performed for three main reasons: there was no suspicion of intraepithelial neoplasia or cancer; cervical, vaginal and vulvar histopathology on pSS women is nonspecific; biopsy is not mandatory for cutaneous involvement in pSS [2, 3].

An analysis of 33 cases identified autoimmune phenomena in 18,2% women with LUs [5]. Close follow-up is recommended to confirm healing and to rule out progression to systemic disease [10]. The described case report shows LUs as a clinical sign of pSS, due to an overexpressed immune response to infection on the presence of a predisposing autoimmune background. Even in cases with documented infection, LU's should be considered as a possible manifestation of many autoimmune/inflammatory disorders, beyond the classic associations such as Behçet's syndrome or Crohn's disease. This can only be achieved with a broad multidisciplinary approach toward individualized care for patients with acute genital ulcers.

## Data Availability

Data supporting our findings can be found by contacting the corresponding author.

## Abbreviations

ANA: Anti-nuclear antibodies
EBV: Ebstein-Barr virus
EULAR: European League Against Rheumatism
FLS: Focal lymphocytic sialadenitis
FS: Focus score
IgG: Immunoglobulin G
LSG: Labial salivary gland
LUs: Lipschütz ulcers
pSS: Primary Sjögren's syndrome
RF: Rheumatoid factor
RNSAGU: Reactive nonsexually related acute genital ulcers
SS: Sjögren's syndrome.
